# Author Index for Volume 92

**DOI:** 10.1038/sj.bjc.6602669

**Published:** 2005-06-14

Aaltonen, LA 1126, 2240

Abdel-Hamid, A-H 815

Abisgold, JD 1307

Abnet, CC 176

Adachi, M 1486

Adachi, Y 1193

Adami, J 1326v

Adamo, V 467

Adams, IP 1493

Adams, RL 1493

Adélaïde, J 382

Adelman, AS 2084

Aebersold, DM 41

Aghcheli, K 176

Aglietta, M 1261

Agnantis, NJ 396

Agostinis, P 1406

Agulnik, M 799

Ahmed, N 113, 1475

Ahn, YC 1226

Aikou, T 284

Akaishi, J 2216

Akamatsu, T 312

Akbulut, H 639

Akino, K 1165

Akiyama, Y 1130

Akizuki, N 2089

Albanese, C 1581

Albers, A 913

Albert, V 1430

Albrektsen, G 167

Alderson, R 1430

Aldridge, SE 1531

Alexopoulos, A 645

Al-Ghnaniem, R 838

Alhopuro, P 1126

Allart, S 747

Allen, DS 1283

Allen, LT 1702

Allen, NE 1283

Allen, SA 147

Allgar, VL 1959, 1971

Almstrup, K 1934

Altermatt, HJ 41

Altinbas, M 639

Altura, RA 359

Alvarez, P 1358

Amano, H 1372

Amaro, T 1892

Andersen, A 1288

Andersen, CL 2240

Anderson, TJ 955

Andersson, S 2195

Andreyev, HJN 1663

Angeletti, C 572

Angerson, WJ 1834

Ansary-Moghaddam, A 2076

Ansell, W 36

Ansorge, W 1934

Antalis, TM 760

Antinori, A 2225

Antoniou, AC 1337

Anzalone, R 888

Apessos, A 396

Appleby, P 838

Arango, D 2240

Archer, CD 475

Ardavanis, A 645

Ardine, M 634

Arends, JJ 445

Arimoto, T 2286

Armstrong, JL 696

Armstrong, N 1524

Armstrong, S 334

Arnold, J 784

Arnold, JM 2024

Arrieta, O 1247

Arslan, A 601

Asaka, S 2216

Asano, T 1486

Ascoli, V 188

Assersohn, LA 475

Astoul, P 13

Astuti, D 1574

Atkin, SL 1493

Atzpodien, J 843

Auer, G 389

Ausems, MGEM 1671

Awada, A 1855

Axelson, H 751

Axelson, M 1467

Azizi, E 2278

Azria, D 2114

Azzabi, A 1006

Azzouzi, A-R 236

Baeuerle, PA 342

Bågeman, E 857

Bagley, S 503

Bai, AHC 2190

Baildam, A 673

Bakhanashvili, M 1881

Bakkevold, KE 1372

Baldelli, AM 1051

Baldi, A 2225

Ball, RY 2171

Balleine, RL 1366

Ballestrero, A 1948

Baltali, E 639

Bamberger, A-M 2206

Bando, H 553

Banks, RE 2140

Barachini, S 681

Baram, Y 1611

Barbosa, AP 1892

Barchielli, A 156, 1815

Bardini, M 1104

Bardwell, H 2160

Barker, G 113

Barlési, F 13

Barlow, C 36

Barnett, Y 1524

Baron, JA 594

Barr, MP 328

Barrass, B 2166

Barrett, JH 2262

Barrett-Lee, P 1869

Bartholomeus, S 1855

Bartlett, JMS 625

Barzi, F 2076

Barzilai, SE 1144

Basolo, F 681

Bass, R 2171

Bassi, C 1372

Bateman, A 1450

Baur, M 1019

Baxevanis, CN 72

Beale, P 832

Beard, JB 278

Bech-Knudsen, F 2240

Beck, A 981

Becker, JC 1398

Bednaruk-Młyński, E 1038

Beech, J 628

Beemer, FA 1671

Beery, E 1881

Belghiti, J 94

Bell, WR 80

Bellelli, S 572

Bellino, R 634

Bellocco, R 1326

Bellone, S 1561

Bellù, F 188

Benencia, F 1182

Benner, A 1746

Bennett, P 60

Bensing, JM 1671

Benson, C 7

Benson, RJ 241

Berardi, R 1051

Berg, K 2004

Berghmans, T 131

Bergkvist, L 1803

Berkhof, J 1388

Berkowitz, RS 983

Berlin, J 1846

Bermejo, JL 162

Bernini, GP 1104

Bernstein, L 1614

Berruti, A 634

Bertetto, O 634

Bertheault-Cvitkovic, F 820

Berthon, P 236

Bertolotto, M 1948

Bhatnagar, AS 1173

Biade, S 1149

Bianco, AR 467, 1644

Bianco, R 1644

Bibeau, F 2114

Biberfeld, P 1467

Bicknell, R 1696

Bidmead, M 488

Biesma, B 15

Bilous, AM 1366

Birch, MA 1531

Birenbaum, M 1881

Birkenkamp-Demtroder, K 2240

Birnbaum, D 382

Bishop, DT 2262

Bisset, M 794

Bissett, JD 1001

Bitossi, R 634

Bjørge, L 895

Blake, P 1663

Bläker, H 1746

Bléchet, C 775

Bleck, JS 1862

Bleeker, MCG 1388

Bleiberg, H 1055

Bliss, JM 1679

Bloom, M 1430

Blot, WJ 594

Bobrow, LG 2201

Böcker, W 1089, 1720

Boddy, AV 696, 1006, 1626

Bode, U 480

Boffetta, P 176, 1288, 1326

Bogdanova, T 1545

Bomanji, JB 1046

Bomstein, Y 1517

Boon, EMJ 1078

Boreham, J 426, 1329

Borg, Å 857

Borgstein, A-M 2032

Borner, M 1055

Borodyansky, L 1581

Boruban, C 639

Borzomati, D 2225

Bosch, FX 770

Bosetti, C 2065

Botelho, T 1892

Botha, JL 2070

Bottini, A 634

Bouchier-Hayes, DJ 328

Boué, DR 359

Boutreux, S 1842

Bouvier, A-M 1842

Boven, E 1636

Bown, S 2004

Boy, D 1948

Boyd, AW 760

Boyd, E 1430

Boyle, P 419

Boyle, RW 1442

Bozzi, F 1984

Bradshaw, TD 350

Bramhall, SR 89

Brammer, RD 89

Brandely, M 1989

Brandsma, D 729

Brechbiel, MW 1069

Bredenkamp, R 2129

Brena, RM 1922

Brendel, E 1855

Brennan, P 176, 1288

Brenner, H 576, 1808

Bressac-de Paillerets, B 2278

Breuhahn, K 935

Brewster, DH 1288, 2095

Bridges, J 102

Brindle, KM 1599

Brink, M 1310

Brischwein, K 342

Brismar, K 1467

Brockbank, EC 102

Brokelmann, M 553

Brouchet, L 743

Brousset, P 743, 747

Brown, JL 722

Brown, M 503

Brown, NS 1696

Brown, TH 1759

Browne, B 120

Bruce, J 225

Bruzzi, P 24

Bucchi, L 156, 1815

Buchen, S 480

Büchler, MW 1372

Buckanovich, RJ 1182

Buckowitz, A 1746

Budroni, M 188

Buemi, A 1842

Buerger, H 1720

Buffoni, L 580

Bugat, R 820

Buhl, L 2240

Buiatti, E 156, 1815

Bukawa, H 1915, 2181

Bukowski, RM 2266

Bulkmans, NWJ 1800

Bulusu, R 1997

Burcombe, RJ 147

Burden, AC 2070

Burgemeister, FC 1366

Burgess, AW 1069

Burnet, NG 241

Burnett, A 1561

Burns, HJG 631

Busnach, G 572

Bussolati, G 1261

Butler, R 60

Büyükcelik, A 639

Byrne, AM 328

Byth, K 1366

Cai, KL 967

Caldas, H 359

Caldes, T 1922

Caldon, LJM 55

Calvert, AH 1006

Calvert, PM 1006

Cameron, D 1869

Camidge, DR 1837

Campbell, B 1358

Campbell, C 376

Campbell, FC 2160

Campbell, I 673

Campbell, NC 1001

Canal, P 820

Cane, S 1561

Canna, K 651

Cannon, MJ 1561

Cao, D 1069

Capela, J 1892

Cappello, F 888

Caraglia, M 1621

Carcenac, M 1442

Caricato, M 2225

Carlomagno, C 1644

Carmichael, J 350

Carrell, J 1430

Carstensen, J 1785

Carter, R 21

Cartwright, G 1069

Carvalho, LP 259

Casanova, M 1984

Cascinu, S 1051

Casper, RF 416

Cassidy, J 1001

Castellani, R 1984

Castellano, P 1644

Castro, J 389

Catalano, G 1644

Catalano, V 1051

Catchpoole, D 1574

Catovsky, D 1352

Catrina, S-B 1467

Cauzig, P 662

Cawkwell, L2185

Cefalo, G 1984

Chaffanet, M 382

Chambers, WA 225

Chan, FKL 2190

Chan, MWY 2190

Chang, CS 1013

Chang, HM 246

Chang, JC 618

Chang, JT-C 30

Chang, T-Y 1906

Chang-Claude, J 2039

Chantrel-Groussard, K 236

Chao, HJ 1013

Charlton, PA 722

Charnley, N 1221

Chatelut, E 820

Chau, I 1352

Chen, C-C 80

Chen, HH-W 1906

Chen, I-H 30

Chen, KY 513

Chen, M 513

Chen, T 873

Chen, VW 2084

Chen, W 1614

Chen, W-F 929

Chen, X 784

Chenevix-Trench, G 784, 2024

Cheng, A-J 30

Cheng, AL 1013

Cheng, H-L 1906

Cheng, JR 2059

Cherian, J 601

Chesser, P 1221

Cheung, Y-B 668

Chia, K-S 1288

Chiari, C 1209

Choi, CW 827

Choi, IK 827

Cholerton, S 1626

Choo, MK 1690

Choudhury, A 1221

Chow, N-H 1906

Christensen, LL 2240

Christensen, M-LM 995

Chu, ESH 2190

Chung, CY 1013

Ciardiello, F 1644

Cigolari, S 1621

Citterio, F 572

Civati, G 572

Clark, J 376

Clarke, CL 1366

Clarke, NW 125, 503

Clarke, OJ 1442

Clarke, S 832

Clavel-Chapelon, F 2042

Clements, JA 760

Coe, BP 1553

Cohen, EEW 1341

Cohen, GM 736

Cohen, JI 1593

Coldman, AJ 961

Coleman, MP 1279, 2095

Coleman, R 1989

Collini, P 1984

Collins, AT 2171

Colucci, G 1621

Condron, CM 328

Conejo-Garcia, JR 1182

Conway, S 1149

Cook, PA 2107

Cooke, TG 631

Cooper, CS 376

Cooper, RA 1221

Cooper, WN 1574

Copland, C 1046

Coppes, RP 539

Coppola, R 2225

Cornen, X 820

Corrie, PG 1997

Coşkun, Ş 639

Costa, L 2266

Costanzo, FD 24

Costanzo, R 467

Coukos, G 1182

Coupland, C 1794

Couvelard, A 94

Cox, A 807

Craig, W 1001

Cranston, D 2140

Cree, IA 1997

Crew, J 2140

Crocetti, E 188

Crow, P 2166

Crowe, P 2160

Crown, J 120

Cruickshank, ME 222

Cuckle, H 955

Cullen, MH 2107

Cummings, J 532

Cummings, M 2024

Cunningham, AP 1337

Cunningham, D 1352, 1650, 1976

Curwen, J 2148

Cussenot, O 236

da Silva, AJ 342

Däbritz, J 405

Dagg, K 1834

Dahan, M 743

Dal Maso, L 188

Daley, FM 147

Dalgleish, AG 792

Damiano, V 1644

Dancey, G 36

Danese, S 634

Danesi, R 681

Dangoor, A 628

Daniel, F 1650

Danielidis, I 396

D’Aprile, M 467

Darnton, AJ 587

Davidson, N 1869

Davidson, SE 449

Davies, E 1201

Davies, FE 217

Davis, L 1846

Dawsey, SM 176

Day, ML 2018

Day, NE 967

Day, RS 47

de Bock, GH 1336

de Bono, J 7

de Bruïne, AP 1310

de Goeij, AFPM 1310

de González, AB 2076

de Graaf, M 882

de Groot, DJA 1459

de Jong, RS 445

de Jong, S 1459

de la Longrais, IAR 634

De Laurentiis, M 467

De Lena, M 467

De Lisi, V 156, 188, 1815

De Placido, S 467, 1644

de Rooij, FWM 1126

de Valeriola, D 1855

De Vita, F 1644

de Vries, EGE 1459

de Wit, NJW 2249

de Witte, PAM 1406

Deapen, D 2095

Dearnaley, DP 488, 1663, 2107

Debatin, K-M 1084

Degano, B 743

Degott, C 94

Deissler, H 231

Dejardin, O 1842

Del Prete, S 1621

Del Tacca, M 681

Delafosse, P 1842

Delisle, MB 747

Delord, JP 820

Delozier, T 1989

Démaret, E 2095

Demirkazik, A 639

Denkert, C 1729

Deri, M 681

Derksen, P 1078

Dervenis, C 1372

Detours, V 1545

Devocelle, M 328

Di Martino, N 1644

Diadema, MR 1644

Diamond, MP 416

Didier, A 743

Diem, E 662

Dienemann, H 1084

Diepstra, JHS 2249

Dieras, V 820

Dietel, M 1729

Dietl, J 981

Dietze, A 2004

Dijkman, R 2032

Din, FVN 1137

Dirix, L 1055

Dittrich, C 1019

Dive, C 449, 532

Djonov, V 41

Doak, SH 1759

Dobrowsky, W 1209

Dobson, C 1430

Doddoli, C 13

Dodson, A 376

Dodwell, D 1869

Dogliotti, L 24, 634

Doll, R 426, 1329

Donadio, M 634, 1261

Donnelly, ET 2160

Dooley, MJ 867

dos Santos Silva, I 1283

Dote, H 1117

Douglas, C 1358

Douglas, DA 320

Douglas, ML 760

Dove, J 147

Dowsett, M 906

Drabkin, HA 2266

Dricu, A 1467

Duffy, AM 328

Duffy, MJ 120

Duffy, SW 3, 597

Dumont, JE 1545

Dunlop, MG 1137

Durkin, J 532

Durrani, O 2153

Durrant, LG 1358

Dusart, M 131

Duyster, J 2129

D’yachkova, Y 961

Dyrskjøt, L 2240

Dziadziuszko, R 1038

Easton, DF 1337

Ecke, M 843

Edwards, B 1430

Edwards, DR 2171

Edwards, S 376

Eeles, R 488

Eggel, M 2018

Egger, M 430

Eggo, MC 89

Eimermacher, A 2122

Ell, PJ 1046

Ellenson, LH 415

Ellis, P 1679

Ellis, V 2171

El-Naggar, AK 1899

Emberton, M 797

Emery, PW 838

Emi, M 2216

Emoto, M 1098

Encío, I 690

Endo, T 1193

Endo, Y 1915

Eng, C 1922

Engel, O 2018

Engholm, G 995

Erkisi, M 639

Errington, F 1450

Escobar, E 1247

Evans, A 949

Evans, BG 194

Evans, HS 194

Evelhoch, JL 1599

Ewertz, M 1293

Fahimi, S 176

Falcini, F 188

Falcone, A 24

Falkmer, U 1506

Fallowfield, L 807, 1393

Fanelli, F 1621

Fangusaro, JR 359

Farchi, F 572

Farris, A 467, 634

Febbraro, A 1621

Federico, M 156, 188, 1815

Feltbower, RG 978

Fenig, E 1881

Fentiman, IS 1283

Ferguson-Smith, AC 1574

Ferrandina, G 271

Ferrari, A 1984

Ferrari, E 1621

Ferraù, F 467

Ferretti, S 156, 188, 1815

Feyerherd, P 1215

Fietkau, R 1215

Filipski, E 1684

Fiscella, M 1430

Fischel, J-L 1063

Fishwick, K 1006

Fitzmaurice, RJ 449

Fitzpatrick, DR 760

Flechtenmacher, C 770

Fleischhack, G 480

Fleshman, JW 259

Flieger, D 2122

Flohr, P 376

Focan, C 1684

Fodstad, Ø 1773

Foeglé, J 459

Foitzik, T 1215

Ford, D 1626

Formato, R 1621

Formento, P 1063

Fornara, P 843

Forrest, LM 1834

Fossa, SD 2107

Fossati-Bellani, F 1984

Foster, C 376

Foster, JR 1837

Foulis, AK 21

Fountzilas, G 396

Fowler, P 1759

Franc, B 1545

France, B 60

Franceschi, S 188, 572, 601, 1591, 2065

Franke, A 1349

Frassineti, G 24

Freathy, C 722

Frederiksen, K 201

Freier, K 770

Friedlander, M 832

Friedman, E 1144, 2278

Frigerio, A 156, 1815

Friis, S 201, 594, 1293, 1302

Frisch, B 1421

Fryzek, JP 1302

Fu, PP 873

Fujii, H 1253, 1486

Fujimori, K 312

Fujimori, M 2089

Fujisawa, T 942

Fujita, H 1754, 2102

Fukino, K 1922

Fukuda, K 562

Fukudo, S 2089

Fukunaga, Y 1486

Funata, N 553

Fuqua, SAW 618

Furihata, K 312

Fusco, A 1817

Gabe, R 597

Gacinovic, S 1046

Gadd, J 488

Galanski, M 1862

Galateau-Salle, F 743

Gale, EA 2070

Galeone, C 2065

Galizia, E 1051

Galizia, G 1644

Gallagher, WM 1702

Galle, PR 2122

Gallo, H 570

Gallotta, V 271

Gan, H 1069

Gandola, L 1984

Gao, YT 2059

Garcia-Manero, G 1165

García-Navarrete, R 1247

Gardner, TA 2233

Garrett, M 7

Garuti, A 1948

Gassler, N 1084

Gatei, M 784

Gatling, W 2070

Gatter, K 94

Gazdar, AF 942, 1117, 1553

Gebbink, M 729

Gebert, J 1746

Geer, T 2122

Geiger, AM 1614

Gemma, A 1711

Gemmill, RM 2266

Generali, D 634

Gentle, D 1574

George, WD 631

Georgoulias, VA 396

Gerçel-Taylor, C 305

Gerritsen, WR 882

Gershoni-Baruch, R 1144

Gertenbach, U 843

Gessi, M 271

Ghadirian, P 971

Giaccone, G 882, 1636

Giacomin, A 188

Giantonio, B 1846

Giddings, I 376

Gil, MJ 690

Gil, T 1855

Gillams, AR 1825

Giotta, G 1621

Giovannetti, E 681

Giovannucci, E 1803

Girard, L 1553

Girnita, A 2097

Girnita, L 2097

Glasgow, SC 259

Glen, P 21

Gnant, M 1655

Gneist, M 1019

Goda, R 1240

Goh, C 668

Goldacre, MJ 1298, 1307

Goldbohm, RA 1310

Goldstein, D 832

Gondos, A 1808

Gonzalez Baron, M 1055

Gorman, A 1702

Gorzegno, G 634

Gosslau, A 513

Göster, P 342

Gotoh, M 1130

Gout, S 838

Gowardhan, B 320

Gracien, E 2122

Graham, JD 2107

Grainge, MJ 1794

Granström, C 1276

Graubard, BI 1787

Gray, A 990

Gray, J 60

Graziano, F 1051

Green, MML 449

Greenman, J 1442, 1588, 2185

Gregory, RK 475

Greiner, RH 41

Grenier, J 2114

Greten, TF 1862

Griffin, MJ 1006

Griffith, GL 60

Griffiths, AP 1759

Griffiths, JR 1599

Grigorian, M 1955

Gritzapis, AD 72

Groen, HJM 15

Groneberg, M 1089

Gross, M 981

Groves, A 1046

Groves, FD 2084

Gruber, G 41

Gruel, Y 775

Gruia, G 1055

Gruis, NA 2032

Grund, D 2206

Grundy, R 1574

Grünler, J 1467

Gschwend, JE 2018

Gu, L 278

Gubbay, O 1927

Guevara, P 1247

Guimard, S 236

Guimbaud, R 820

Gunnarsson, C 547

Guo, L 873, 1794

Guo, W 1927

Gustavsson, I 891

Gustmann, C 1720

Guyétant, S 775

Gyllensten, U 891, 2195

Haase, CG 1855

Haba, R 1231

Hachisuga, T 1098

Hackett, C 1663

Haeusler, J 1159

Hagiwara, A 562

Hague, A 736

Haisma, HJ 882

Hakulinen, J 895

Hakulinen, T 576

Halbert, G 1358

Hale, J 1358

Hall, E 488

Hall, M 1702

Haller, A 131

Haller, DG 1846

Hamaguchi, T 1240

Hamana, K 2181

Hamburger, AW 140

Hamdi, M 413

Hamilton, TC 1149

Hanawa, M 1253

Hanby, AM 613

Hänfler, J 405

Hankins, M 990

Hanley, RS 2153

Hansen, S 167

Hao, C 942

Harach, HR 1892

Harari, PM 1819

Harland, SJ 2107

Harmey, JH 328

Harper-Wynne, C 1976

Harrington, K 1450

Harrington, KJ 1663

Harriott, P 328

Harris, AL 1, 94, 1696, 2140

Harris, J 194

Harris, N 838

Harris, SM 722

Harrison, DJ 1268

Hart, CA 503

Hartley, JA 1997

Hart-Prieto, M 2166

Harvie, MN 673

Hasan, C 480

Hasan, F 736

Hasebe, T 847

Hasegawa, K 1486

Haslett, C 522

Hauptmann, S 1729

Hausmaninger, H 1655

Hawkins, RE 1650

Hayes, J 359

Hayward, NK 2032

He, XZ 1321

HeCOG 396

Hédelin, G 459

Heflich, RH 873

HeHeGI 396

Heike, M 2122

Helfman, DM 1955

Hellqvist, E 547

Helms, G 231

Hemminger, G 2206

Hemminki, K 162, 1276, 1288

Henderson, L-J 1553

Hendlisz, A 1855

Hendry, JH 125

Heng, D 1382

Henley, NC 631

Henne-Bruns, D 921

Hennig, M 2129

Henze, G 480

Herbert, C 1842

Herbst, F 1209

Herkommer, K 1159

Hermans, J 1767

Herr, I 1084

Hess, J 41

Hetherington, JW 1588

Heuch, I 167

Hewer, A 2160

Heynemann, H 843

Hickish, T 1976

Hicks, DJ 736

Hicks, RJ 655

Hildenbrand, R 1398

Hill, ADK 120

Hill, M 1352, 1976

Hill, ME 1650

Hillier, SG 1927

Hilmy, M 625

Hilsenbeck, SG 618

Hinoda, Y 1165, 1193

Hinrichs, B 1720

Hinterhuber, G 662

Hirakawa, K 1110

Hiroshima, K 942

Hisatomi, H 1942

Hlushchuk, R 41

Ho, Ci-T 513

Ho, C-L 1906

Hobohm, U 421

Hochhaus, A 1398

Hodgson, JT 587

Hoegel, J 1159

Hoei-Hansen, CE 1934

Hoeller, C 662

Hoermann, K 913

Hofbauer, F 1655

Hofele, C 770

Hofer, MD 2018

Hofsli, E 1506

Hoft, S 207

Hogan, A 2171

Hogewoning, CJA 1388

Höhler, T 2122

Hole, DJ 631

Holloway, MP 359

Holly, JMP 1283

Holmes, M 815

Holzberger, P 1655

Honda, S 1942

Hong, A 1869

Hong, TS 1819

Hoper, M 2160

Horai, T 1877

Horichi, N 1486

Horiguchi, S 553

Horiike, A 1877

Horiuchi, S 1098

Horsman, MR 1599

Horwich, A 488, 1352

Hosaka, S 312

Hoshino, Y 1130

Hosokawa, T 2089

Hosokawa, Y 562

Hotfilder, M 705

Houlgatte, A 236

Howell, A 673

Hsieh, L-L 30

Hsu, CH 1013

Hsu, P-Y 1906

Hu, SP 967

Huang, C 1231

Huang, CF 1321

Huang, J-Y 80

Huang, K-C 983

Huang, TC 1013

Huang, T-H 30

Hubé, F 775

Huddart, RA 488

Hudec, M 1019

Hudson, R 1442

Huggard, PR 2024

Hughes, AM 1837, 2148

Hughes, AN 1006

Hughes, S 1046

Humphreys, R 1430

Hunt, DP 241

Hunter, RD 449

Hussien, MMI 1524

Hutchison, GJ 449

Hutsebaut, N 1545

Huwendiek, S 389

Huxley, R 2076

Hydes, N 1046

Iaffaioli, RV 1621

Ianniello, G 1621

Iannino, N 131

Ibrahim, K 1393

Icli, F 639

Iizasa, T 942

Im, Y-H 1850

Imai, K 1165, 1193

Imoto, S 847

Imrie, CW 21

Incoronato, P 1621

Infusino, S 1644

Inoue, H 1754

Inoue, M 182

Iochmann, S 775

Ishida, T 1165

Ishigami, S 284

Ishihara, T 2134

Ishii, G 847

Ishikawa, S 1231

Ishikawa, T 1110

Ishikawa, Y 1877

Islami, F 176

Isoe, T 1486

Issa, J-PJ 1165

Ito, K 562, 1538, 2216

Ito, Y 562

Iversen, OE 895

Iveson, T 1976

Iwamoto, R 1737

Jack, RH 1201

Jackson, A 1599

Jacobi, CE 1336

Jager, MJ 2032

Jagoditsch, M 1655

Jakesz, R 1209, 1655

Jallo, G 414

Jamieson, NB 21

Jarva, H 895

Järvinen, H 2240

Jassem, J 1038

Jasser, SA 1899

Jayson, GC 1599

Jech, B 1209

Jeekel, H 1372

Jefferies, SJ 241

Jekimovs, CR 784

Jeng, M-H 2233

Jenkins, D 1794

Jenkins, GJS 1759

Jenkins, V 807

Jensen, JL 2240

Jensen, TS 895

Jentsch, H 843

Jentsch-Ullrich, K 1349

Jernström, H 857

Jhavar, S 376

Ji, J 1276

Jiang, Y 359

Jin, YT 1321

Jodrell, D 522

Joel, SP 2140

Johansson, B 2195

Johns, L 949

Johns, TG 1069

Johnsen, SP 1302

Johnson, J 1794

Johnson, KC 971

Johnson, L 1679

Johnson, R 1430

Johnson, SW 1149

Johnston, PG 1524

Johnston, SRD 475

Jonasson, JG 1288

Jones, A 1696, 1989, 2140

Jonson, A 1997

Joos, S 770

Joosens, E 1055

JPHC Study Group 182

Juarez-Brito, MA 2018

Judson, IR 1599

Juhola, M 2240

Jung, C 2233

Jung, CW 1226, 1850

Junginger, T 2122

Jürgens, H 705

Juriens, J 382

Kaaks, R 2042

Kaalhus, O 2004

Kabasawa, K 1033

Kakizoe, T 1240

Kallimanis, G 396

Kamangar, F 176

Kameyama, K 1231

Kampinga, HH 539

Kanakaraj, P 1430

Kandylis, C 645

Kang, D 1273

Kang, HJ 246

Kang, JH 1226, 1850

Kang, WK 1850

Kang, Y-K 246

Kanzaki, N 1130

Kao, C 2233

Kao, Y-L 80

Karhu, A 1126

Karner-Hanusch, J 1209

Kashiki, Y 2102

Katajisto, P 1126

Kataoka, K 1240, 1711

Kato, H 2134

Kato, M 1915

Kato, S 2286

Katsaros, D 634

Katsura, K 562

Katsuyama, T 312

Kaufman, JC 1581

Kawarabayashi, T 1098

Kawasaki, N 1033

Kayani, I 1046

Kaye, S 7

Kaye, SB 2107

Kayitalire, L 1684

kConFab Investigators 784

Ke, H-S 80

Keen, H 2070

Keikavoussi, P 1398

Kelley, MR 334

Kelly, C 1006

Kendall, C 2166

Kenna, T 1702

Kennedy, MJ 1813

Kersting, C 1720

Kesson, E 631

Key, TJ 1283

Khanna, KK 784

Khoo, K-S 668

Khoo, V 1663

Kido, S 252

Kienle, P 1746

Kiesel, L 1720

Killoran, J 1702

Kiltie, AE 2262

Kim, BS 246, 827

Kim, C-H 334

Kim, C-S 1273

Kim, EJ 1955

Kim, GG 913

Kim, JS 827

Kim, K 1226, 1850

Kim, R-S 2233

Kim, S 1899

Kim, SJ 827

Kim, ST 1850

Kim, TW 246

Kim, WS 1226, 1850

Kim, YH 827

Kin, S 562

Kirchhoff, T 1862

Kishikawa, T 1737

Kitahara, K 252

Kitajima, Y 252

Kittler, H 662

Kiyono, T 290

Klautke, G 1215

Klebanoff, MA 1787

Klein, G 2010

Kleisbauer, J-P 13

Klempnauer, J 1862

Klevesath, MB 2201

Kliewer, EV 1288

Klingel, K 2010

Klinkenbijl, JHG 1372

Kloor, M 1746

Knaebel, H-P 1746

Knopp, MV 1599

Ko, YH 1226

Kobayakawa, M 2089

Kobayashi, A 2134

Kobayashi, H 1737

Kobayashi, M 1782

Köbel, M 1729

Koehler, T 2039

Koenigsmann, M 1349

Kogure, M 1130

Koide, K 562

Koike, H 1538

Koizumi, K 1690

Kokubo, Y 1711

Komurcu, S 639

Kon, T 1414

Kono, K 1253

Korch, C 2266

Koretz, K 231

Kornmann, M 921

Kosmidis, PA 396

Kovács, AF 206

Kovacs, G 376

Kovarikova, M 1078

Kranenburg, O 729

Kreienberg, R 231

Kreuser, E-D 1989

Krewski, D 971

Kristen, P 981

Krol, A 1414

Kronenwett, U 389

Kropp, S 2039

Kruhøffer, M 2240

Kubicka, S 1862

Kubrak, J 1038

Kuczyk, M 2010

Kudoh, S 1711

Kuefer, R 2018

Kumar, DM 1393

Kuo, C-C 80

Kuroda, M 290

Kusnierczyk, W 1506

Kvåle, G 167

Kypridis, A 1069

La Rocca, G 888

La Rosée, P 1398

La Vecchia, C 2065

Labrousse, F 747

LaCasse, E 532

Laegreid, A 1506

Lafitte, J-J 131

Lahav, M 1881

Lai, C-H 30

Lai, PBS 1268

Laiho, P 2240

Lain, S 434

Lainakis, G 645

Laing, SP 2070

Lam, S 1553

Lam, T 1588

Lam, WL 1553

Lane, DP 434

Langaas, M 1506

Langdon, SP 1927

Larriba, MJ 985

Larsson, O 2097

Larsson, SC 1803

Latif, F 1574

Latil, A 236

Latumalea, SP 539

Laub, PB 1149

Laud, K 2278

Laud, PJ 1837

Launonen, V 1126

Launoy, G 1842

Laurberg, S 2240

Lauria, R 467

Lauriola, L 271

Law, GR 978

Lawrence, D 488

Le, TKP 1459

Le Monnier, K 2140

Leach, MO 1599

Lebitasy, MP 459

Lee, EHL 1553

Lee, F-T 1069

Lee, J 1226, 1850

Lee, J-S 246

Lee, SA 2049

Lee, S-H 334, 1226, 1850

Lee, SI 1226

Lee, S-J 2233

Lee, SM 628

Lee, SS 1226

Leek, R 94

Lefebvre, C 532

Leffers, H 1934

Legge, F 271

Legood, R 990

Lehtonen, R 1126

Leigh, IM 1953

Lemarié, E 775

Lennard, TWJ 1531

Lentjes, MHFM 1310

Leong, S-S 1382

Leong, T 655

Lersch, C 2129

Letessier, A 382

Leung, HY 320

Leung, WK 2190

Lévi, F 1684, 2065

Lewandowska, A 1038

Lewensohn- Fuchs, I 2195

Lewis, I 1358

Lewitt, M 1467

Leys, MBL 445

Li, B 967

Li, C 80

Li, C-Y 1414

Li, DR 967

Li, F 212

Li, X 1288

Li, XM 1684

Li, Y 929

Liao, C-T 30

Libertino, JA 2153

Lichter, P 770

Lieto, E 1644

Lilla, C 2039

Lima, J 1892

Limite, G 467

Lim-Joon, D 655

Lin, JF 1013

Lin, Y-J 1906

Lind, M 815

Lind, MJ 1006

Lindelöf, B 1326

Lindow, SW 1493

Liou, S-H 30

Lishner, M 1517

Litvinov, SV 1767

Liu, D 1231

Liu, H-S 1906

Liu, S 607, 1414

Liu, Y 921

Liu, Z 1069

Lochon, I 820

Loehrer, PJ 2233

Loi, S 655

Lokiec, F 820

Longerey, B 1989

Longnecker, MP 1787

Löning, T 2206

Lopez-Crapez, E 2114

Lordick, F 2129

Lorenz, R 981

Lorenzen, S 2129

Lorusso, V 467, 634

Lothaire, P 131

Lu, XZ 967

Lu, YS 1013

Luben, RN 967

Lucas, CD 522

Ludwig, K 1215

Lukanidin, E 1955

Luksch, R 1984

Lund, T 1773

Luria, D 1881

Lutze, G 1349

Ma, H 2153

Maarouf, N 1842

MacAulay, C 1553

Macdonald, L 225

MacFarlane, M 736

Mackay, J 655

MacKinnon, AC 522

Madden, L 1442

Maddineni, SB 125

Maenhaut, C 1545

Maesawa, C 1130

Magistrelli, P 2225

Mahé, C 601, 1591

Maher, ER 1574

Mahoney, A 1430

Maier, A 1209

Maier, C 1159

Maissi, E 990

Maitland, NJ 2171

Maitra, SK 570

Mäkelä, TP 1126

Makris, A 147

Małachowski, K 1038

Malacrino, C 2225

Malekshah, AF 176

Malekzadeh, R 176

Mandal, M 1899

Manetopoulos, C 751

Manns, MP 1862

Mano, M 1855

Mansi, J 1869

Manton, KJ 760

Manzione, L 467, 1621

Marano, O 1621

Maraveyas, A 815, 1006, 1588

Marchianò, A 1984

Margison, GP 125

Mari, D 1051

Marian, C 2278

Markmann, I 1349

Marshall, CJ 102

Marteau, TM 990

Martin, B 131

Martin, SG 350

Martinelli, E 271

Martínez-Merino, V 690

Martos, C 1288

Marzano, N 1621

Mascaux, C 131

Maskow, A 843

Mason, T 1869

Massambu, C 1467

Massey, T 1201

Massimino, M 1984

Masuda, T 1130

Masuya, D 1231

Mataki, Y 284

Matano, E 1644

Matecka-Nowak, M 1038

Mathers, ME 320

Matsubara, T 2102

Matsuda, K 1711

Matsumoto, K 1026

Matsumoto, M 284

Matsumoto, Y 2286

Matsumura, Y 1240

Matsuo, M 1690

Matsuyama, S 252, 1130

Mattern, J 1084

Mattsson, B 1720

Maune, S 207

Máximo, V 1892

Maxwell, RJ 1599

Mayer, A 1997

McArdle, CS 651, 1834

McArdle, PA 651

McBride, M 1288

McCaffery, K 265

McCluggage, WG 2160

McCracken, SRC 320

McDermott, E 120

McElvenny, DM 587

McGlynn, KA 1787

McIntyre, D 1599

McKay, CJ 21

McKee, RF 651

McKie, AB 320

McKinney, PA 978

McLaughlin, CC 2084

McLaughlin, JK 201, 594, 1293, 1302

McLeod, HL 259

McMillan, DC 21, 625, 651, 1834

McNicol, A-M 651

McNulty, H 1524

Mead, GM 2107

Meazza, C 1984

Mecklin, J-P 2240

Meert, A-P 131

Mei, N 873

Meijer, CJLM 601, 1388, 1800

Mekada, E 1737

Melcher, A 1450

Mellemkjaer, L 201, 1288, 1293

Melton, RG 480

Menachem, TD 1144

Mendes, R 475

Menges, M 2122

Meri, S 895

Merrick, A 1450

Mery-Mignard, D 820

Mesters, R 1720

Meter, A 539

Mey, V 681

Michael, M 655, 832

Mihalatos, M 396

Milani, M 634

Milano, G 1063

Milde-Langosch, K 2206

Mileshkin, L 867

Miller, ID 222

Mills, GB 1899

Milward, K 1450

Mimori, K 1754

Mimura, K 1253

Minegishi, Y 1711

Minetti, E 572

Minna, JD 1553

Mints, M 2195

Mischinger, HJ 1655

Mistry, P 722

Mita, H 1165

Mitchell, A 1393

Mitchell, P 655, 832

Mitragotri, S 499

Mitsui, F 1253

Mittlböck, M 1655

Mitwally, MF 416

Miyagawa, K 562

Miyagawa, N 1253

Miyamoto, S 1737, 2216

Miyazaki, K 252

Miyoshi, A 252

Mizutani, K 2216

Mlineritsch, B 1655

Moberg, M 891

Moehler, M 2122

Moeslein, G 1126

Mohren, M 1349

Moinzadeh, P 935

Mok, SC 983

Møller, H 194, 201, 570, 1201

Montemurro, F 1261

Morant, R 1055

Moretti, A 1104

Morgan, GJ 217

Mori, K 1130

Mori, M 1754

Morofuji, N 562

Morré, DJ 690

Morris, AD 2070

Morris, CD 2148

Morrison, E 1450

Moss, S 949, 955, 990

Mouri, Z 820

Mozziconacci, M-J 382

Mross, K 1989

Mukai, K 290

Mukesh, B 655

Mukherjee, A 350

Müller, C 2004

Müller, CA 2010

Müller, D 1499

Müller, J 342

Müller, V 2206

Muñoz, A 985

Munro, AJ 434

Murai, M 1165

Murakami, M 1117

Murati, A 382

Murphy, A 55

Murphy, R 1869

Mutter, GL 1922

Myers, EN 913

Myers, JN 1899

Näätsaari, L 1126

Nagabhushan, N 1046

Nagahara, H 1754

Nagao, K 1942

Nagao, Y 2102

Nagasaka, K 2286

Nagata, C 2102

Naito, M 1117

Nakagawa, K 1877

Nakagawa, S 2286

Nakamura, H 1033

Nakamura, I 1240

Nakamura, K 1486, 2134

Nakano, H 1737

Nakase, Y 562

Nakashima, T 1231

Nakatomi, I 1240

Nakaya, N 2089

Narayansingh, GV 222

Nasioulas, G 396

Nasrollahzadeh, D 176

Nasti, G 1621

Natsugoe, S 284

Nazroo, J 265

Neal, KR 1794

Neal, RD 1959, 1971

Negri, E 580, 2065

Nei, T 2286

Nencioni, A 1948

Neoptolemos, JP 1372

Netzel-Arnett, S 760

Newnham, A 194

Ng, EKW 2190

Ng, G-Y 668

Ng, L 2153

Ng, M 1352

Ng, S-K 983

Ng, S-W 983

Ngampolo, D 480

Ngan, SYK 655

Ni, X 983

Nicol, DL 760

Nicolson, M 1976

Nicolson, MC 1001

Niedre, MJ 298

Nielsen, JE 1934

Niesporek, S 1729

Nishino, Y 2089

Nishio, K 1877

Nishio, M 1877

Niv, E 1517

Niven, D 1927

Nix, P2185

Nkondjock, A 971

Noble, S 147

Nobuhara, Y 1110

Noel, S 131

Nordenberg, J 1881

Norman, A 1663

Norman, AR 1352, 1650, 1976

Noro, R 1711

Nortier, JWR 445

Nosho, K 1193

Noske, A 1729

Nouraie, M 176

Novogrodsky, A 294

Nupponen, NN 2240

Nurgat, ZA 1001

Nuttall, M 797

Nuttall, RK 2171

Nutting, CM 488

Nuzzo, G 2225

Oates, J 1650, 1976

O’Brien, TJ 278

Ochiai, A 847

Odenbro, Å 1326

O’Donnell, D 1450

O’Donovan, N 120

O’Dwyer, PJ 1149, 1846

O’Dwyer, ST 430

Oettle, H 405

Ogasawara, S 1130

Ogawa, K 1754

Ogawa, T 553

Ogawara, K 2181

Ogisawa, K 1110

Oh, SC 827

Oh, ST 246

Ohayon, T 1144

O’Higgins, N 120

Ohmori, T 1486

Ohtani, S 1130

Ohyanagi, F 1877

Ohyashiki, JH 1942

Ohyashiki, K 1942

Oikawa, K 290

Oishi, H 2286

Okamoto, J 2216

Okano, T 1711

Okumura, H 284

Okumura, S 1877

Old, LJ 1069

Oliva, K 113, 1475

Oliver, RTD 36

Olivotto, IA 961

Olopade, FA 1663

Olsen, JH 201, 1293

Olsen, ØE 1773

Olsson, H 857

Onda, M 2216

Onda, T 1026

Ong, Y-K 1382

Onoda, N 1110

Onur, H 639

Ooi, A 1253

Oosterhoff, D 882

Ord, JJ 2140

Orditura, M 1644

Oriandini, C 1104

Ormerod, MG 906

Ørntoft, TF 2240

Osborne, MR 2160

O’Shea, C 120, 1702

O’Shea, DF 1702

Oshima, A 2095

Ota, T 1117

Otake, N 1486

Otani, T 1782

other Goim authors 1621

O’Toole, D 94

Ottonello, L 1948

Out, C 2032

Ou-Yang, T 967

Overmeer, RM 882

Oyama, K 1130

Oyama, T 1538

Paci, E 156, 1815

Packer, L 2032

Padhani, AR 1599

Paesmans, M 131

Pagliarulo, C 467

Pagonis, C 1358

Paiss, T 1159

Palazzo, S 467

Paliwal, S 499

Palmas, A 580

Palmeri, S 467

Palmieri, M 1561

Pals, ST 1767

Pandha, H 1450

Panousis, C 1069

Papa, MZ 1144

Papamichail, M 72

Papendorf, F 1862

Pappagallo, G 1621

Paradiso, A 467

Paraskeva, C 736

Park, JO 1850

Park, K 1226, 1850

Park, KH 827

Park, KW 1226, 1850

Park, SH 1226

Park, S-J 334

Park, SK 1273

Park, YH 1226

Park, YS 1850

Parkin, DE 222

Parkin, DM 1808

Parry, A 1626

Parry, EM 1759

Parry, JM 1759

Parslow, RC 978

Paschka, P 1398

Patmore, H 2185

Patriarca, S 156, 188, 1815

Patrone, F 1948

Patterson, CC 2070

Patterson, MS 298

Patya, M 294

Paul, AB 2262

Pavey, S 2032

Pavlotsky, F 2278

Pearson, ADJ 696, 1626

Pecorelli, S 1561

Pedersen, L 1293

Pedicini, T 1621

Pehamberger, H 662

Peintinger, F 982

Pèlegrin, A 1442

Pelucchi, C 580, 2065

Peng, Q 2004

Pennington, CJ 2171

Pepe, S 1644

Perez, SA 72

Persad, R 2166

Peschel, C 2129

Pestell, RG 1581

Peters, M 342

Peters, WP 47

Peto, J 587, 1283

Peto, R 426

Petrella, G 467

Pezzella, F 94

Pezzella, G 1621

Pfeffer, MR 1611

Pharoah, PDP 1337

Phillips, DH 2160

Phillips, N 961

Piccart, M 1855

Picciocchi, A 2225

Pierga, JY 820

Pieterse, AH 1671

Piffer, S 188

Pike, MC 2049

Pinder, SE 2201

Pineda, B 1247

Pinedo, HM 882

Piolatto, PG 580

Pira, E 580

Pisano, A 1621

Pisconti, S 1621

Piselli, P 572

Pistillucci, G 467

Pittam, M 147

Piva, L 1984

Pizza, C 1621

Plass, C 1922

Pliszka, A 1038

Plummer, ER 1006

Plummer, M 601

Podda, M 1984

Poetter, R 1209

Pokrajac, B 1209

Polastri, D 1984

Polesel, J 188

Pommepuy, I 747

Pompe-Kirn, V 1288

Ponder, BAJ 967

Poole, K 1499

Postmus, PE 15

Poulsen, AH 1293, 1302

Pourshams, A 176

Pozzetto, A 572

Prall, F 1215

Prang, N 342

Preithner, S 342

Preston, R 405

Preto, A 1892

Price, L 1626

Price, MJ 587

Price, P 1599

Prior, T 791

Pritchard-Jones, K 1358

Prokopchuk, O 921

Pufulete, M 838

Pukac, L 1430

Pukkala, E 1288

Purohit, A 459

Purushotham, AD 2201

Qiao, Z 2070

Quattrin, S 1621

Quinn, M 1475

Quinn, MA 113

Quoix, E 459

Rabinovich, GA 1188

Rachet, B 1279

Rae, MT 1927

Rajkumar, R 601

Rajpert-De Meyts, E 1934

Rakhshani, N 176

Ramm, GA 2024

Randall, KR 1837

Ranelletti, FO 271

Ranson, M 532, 628

Rao, S 1650, 1976

Rappa, F 888

Rathbone, R 1599

Ravagnani, F 1984

Ravaioli, A 156, 1815

Rawal, R 162

Rayzman, VM 1069

Razis, E 396

Redfern, CPF 696

Reed, JA 55

Reed, MWR 55

Régina, S 775

Reijerkerk, A 729

Reiners, C 981

Reitsamer, R 982

Reitz, M 843

Remmel, E 231

Renehan, AG 430

Renner, C 1069

Rennie, JA 838

Reshef, H 1881

Reverdiau, P 775

Reynolds, JV 1813

Rezza, G 188, 572

Riccobene, T 1430

Rice, G 1475

Rice, GE 113

Richard, DJ 784

Richman, PI 147

Riddick, ACP 2171

Rieck, G 2206

Ried, T 389

Rieger-Christ, KM 2153

Rigatos, G 645

Righi, L 1261

Rijntjes, J 2249

Riley, C 113, 1475

Ring, AE 906

Rinkes, IHMB 729

Rischin, D 655

Ritter, MA 1819

Rittgen, W 1398

Rittman, T 1794

Roberts, D 1149

Roberts, ISD 2140

Roberts, JT 2107

Robertson, K 334

Robinson, D 570, 1201

Robson, CN 320

Rocco, I 1948

Roddam, AW 1283

Roemen, GMJM 1310

Roffel, AF 539

Rogers, MA 2140

Rohrbach, F 1421

Roka, F 662

Rollin, J 775

Roman, JJ 1561

Ronckers, C 1787

Roskams, T 1406

Rose, A 2148

Ross, JA 1268

Ross, PJ 1650

Ross, RK 2049

Rosso, R 24

Roth, A 1055, 1421

Rowland-Payne, C 570

Roy, R 1393

Rozendaal, L 1800

Ruan, ZX 2059

Rubin, MA 2018

Ruijter, R 1636

Ruiter, DJ 2249

Ruiz, M 696

Russell, ST 876

Rustin, GJ 1599

Ruszniewski, P 94

Ryan, B 120

Ryu, M-H 246

Saadatian-Elahi, M 176

Sabatier, J 747

Sadjadi, AR 176

Sadler, CJ 1837

Safari, H 2195

Sahai, E 102

Saidi, F 176

Saiki, I 1690

Sainsbury, R 1175

Saisho, H 2134

Saito, K 1130

Saito-Nakaya, K 2089

Sak, A 1089

Sak, SC 2262

Sak, SD 639

Sakakura, C 562

Sakurai, H 1690

Salahi, R 176

Salcedo, T 1430

Salisbury, ELC 1366

Salovaara, R 2240

Salvetti, A 1104

Sandbank, J 1881

Sandberg, T 857

Sanders, TA 838

Sandvik, AK 1506

Sangar, VK 125

Sankaranarayanan, R 601

Santin, AD 1561

Santini, D 2225

Santoro, M 1817

Sapino, A 1261

Sarobba, MG 467, 634

Sasaki, S 847

Sasaki, Y 1165

Sashida, G 1942

Sathyanarayana, UG 942

Satoh, A 1165

Satoh, Y 1877

Sauer, G 231

Saunders, MP 430

Sauvanet, A 94

Sawada, T 1922

Sawasaki, T 278

Scambia, G 271

Scarffe, JH 628

Scarpa, S 2225

Scartozzi, M 1051

Scélo, G 1288

Schaberl-Moser, R 1655

Schadendorf, D 1398

Schaefer, C 913

Schernhammer, E 1019

Schick, J 1149

Schippinger, W 1655

Schirmacher, P 935

Schmid, R 1209

Schmitt, E 843

Schnabel, PA 1084

Schramel, FMNH 15

Schuber, F 1421

Schwartz, B 1855

Scope, A 2278

Scott, AM 1069

Scott, HR 1834

Scott, NW 225

Sculier, J-P 131

Seagroatt, V 1298, 1307

Sebag-Montefiore, D 1221

Sehgal, A 1201

Sehmbi, R 1997

Sehouli, J 1729

Seike, M 1711

Seiwert, TY 1341

Seki, N 1915

Selbo, PK 2004

Selby, P 1450

Semnani, S 176

Senß, A 705

Sencan, O 639

Senesse, P 2114

Senkus-Konefka, E 1038

Senler, FC 639

Seo, JH 827

Serin, D 1989

Serraino, D 188, 572

Seth, R 1794

Sethi, T 522

Sethia, KK 2171

Sevelda, P 1019

Sewram, V 176

Seymour, JF 867

Sgouros, J 815

Shackney, SE 47

Shamash, J 36

Shannon, WD 259

Sharma, M 222

Sharp, L 222

Sharrard, M 503

Shaughnessy Jr, J 1561

Shaw, J 1997

Shen, L 1165

Sheng, H 2233

Shepard, L 1430

Sherlock, DJ 628

Shi, J 607

Shi, Y-Y 929

Shibuya, C 2102

Shibuya, D 2089

Shibuya, M 1711

Shieh, B 80

Shigemasa, K 278

Shigematsu, H 942

Shiiba, M 1915, 2181

Shimada, K 1915

Shimizu, H 2102

Shimizu, K 1240

Shimizu, N 1117

Shimodaira, S 312

Shimomura, K 562

Shimonishi, T 252

Shin, A 1273

Shin, HR 1273

Shin, SW 827

Shipley, J 376

Shirane, M 1130

Shirouzu, K 1754

Shivapurkar, N 942

Shu, XO 2059

Shukla, CJ 2171

Shults, J 1846

Sibaud, D 1055

Siebler, J 2122

Silva, RR 1051

Simeone, E 1644

Simony-Lafontaine, J 2114

Singer, JW 1729

Singh, V 359

Siu, LL 799

Sjölund, J 751

Skakkebæk, NE 1934

Skriver, MV 594

Slater, SD 2070

Slavin, MA 867

Sleeboom, HP 445

Smartt, H 736

Smit, EF 15

Smit, JM 445

Smith, D 1650

Smith, FM 1813

Smith, GW 651

Smith, IE 475, 906

Smith, K 1442

Smith, PD 1837

Smith, WCS 225

Smyth, FE 1069

Snijders, PJF 601, 1388, 1800

Soames, AR 1837

Soares, P 1892

Sobrero, A 24

Sobrinho-Simões, M 1892

Sobue, T 1782

Sohn, HJ 246

Sokal, MPJ 2107

Sondermann, P 705

Song, DH 1581

Songun, I 1767

Sonoda, K 1737

Sørensen, FB 2240

Sørensen, HT 594, 1293, 1302

Sorré, C 1655

Sotelo, J 1247

Sotoudeh, M 176

Spanswick, VJ 1997

Speiser, P 1019

Spence, RAJ 1524

Spendlove, I 1358

Spierings, DCJ 1459

Spreafico, F 1984

Spurdle, AB 784

Stafford, N 2185

Stål, O 547

Stanczyk, FZ 1787

Starink, TM 1388

Stark, LA 1137

Stark, M 2032

Steer, TE 1830

Steger, G 1655

Steiert, I 2010

Steiger, C 342

Stenning, SP 2107

Stephens, RB 1813

Stevens, MFG 350

Stevenson, JP 1149

Stevenson, M 2160

Stewart, L 811

Sticht, C 770

Stiefel, C 41

Stieler, J 405

Stift, A 1209

Stigt, JA 15

Stillman, F 1339

Stillman, FA 1179

St-Jean, M 532

Stocken, DD 1372

Stockhausen, M-T 751

Stoeber, K 1177

Stokke, T 1773

Stone, N 2166

Storm, H 2095

Storm, HH 995

Stracci, F 188

Stratford, IJ 449

Streeter, E 2140

Streeter, EH 1696

Strickland, PT 176

Strumberg, D 1855

Strunz, K 231

Studer, U 41

Stueben, G 1089

Stuschke, M 1089

Stützer, H 935

Sucker, A 1398

Sudo, K 2134

Sudo, T 1754

Sugai, H 1253

Summerhayes, IC 2153

Sumpter, K 1976

Sun, J 1684

Sun, W 1846

Sun, W-S 929

Sundaram, J 499

Sung, C 1430

Sung, JJY 2190

Sutherland, I 426

Sütterlin, M 981

Suzuki, H 1165

Suzuki, K 1538

Suzuki, M 942, 1240

Sweet, K 1922

Swerdlow, AJ 2070

Swindell, R 628

Syed, R 1046

Symonds, RP 1393

Tadenuma, H 2134

Tagliabue, G 188

Taguchi, M 1033

Tait, D 1663

Takada, T 1372

Takagane, A 1130

Takahashi, T 942

Takaku, T 1942

Takao, S 284

Takashima, T 1110

Takatori, H 284

Taketani, Y 1026, 2286

Takezawa, Y 1538

Takizawa, S 2286

Talamini, R 2065

Talmi, YP 1611

Tan, E-H 1382

Tanaka, K 1684

Tanaka, Y 1737

Tanimoto, H 278

Tanzawa, H 1915, 2181

Tao, MH 2059

Taurino, C 1104

Taveira, A 1892

Taylor, DD 305

Tchouhadjian, C 13

te Velde, EA 729

Tebbutt, N 1976

Tehard, B 2042

Teillac, P 236

Teleky, B 1209

ten Bokkel Huinink, D 445

Teng, R-H 80

te-Poele, R 376

Terashima, M 1130

Terenziani, M 1984

Terinde, R 231

Tesselaar, MET 445

Testa, E 1051

Thatcher, N 673

the Canadian Cancer Registries Epidemiology Research Group 971

The GenQuest research team 60

the SCREENREG Working Group 156, 1815

Thedieck, C 2010

Thézenas, S 2114

Thirion, A 2114

Thomas, B 949

Thomas, G 1545

Thomas, GA 2160

Thomas, I 949

Thomas, M 1561

Thommesen, L 1506

Threlfall, T 2095

Thumboo, J 668

Thuret, R 236

Thursky, KA 867

Thykjaer, T 2240

Tian, X 278

Tierney, J 811

Tilby, MJ 1626

Timmer, T 1459

Tio, J 1720

Tisdale, MJ 711, 876

To, K-F 2190

Todd, R 1006

Tofts, PS 1599

Toh, C-K 1382

Toi, M 553

Tokino, T 1165

Tomé, WA 1819

Tomlinson, IP 1126

Tonini, G 2225

Törnberg, S 1785

Torre, J-P 13

Tortoriello, A 1621

Toyooka, S 1117

Toyota, M 1117, 1165

Tozer, GM 1599

Tracey, E 1288

Treasure, FP 241

Trémoulet, M 747

Triantafillidis, JK 396

Tronko, MD 1545

Tryfonopoulos, D 645

Tsai, Y-C 80

Tsubono, Y 1782, 2089

Tsugane, S 182, 1782

Tsuji, I 2089

Tsujioka, H 1098

Tsukuda, K 1117

Tudor-Edwards, R 60

Tufail-Hanif, U 522

Tumino, R 188

Turbiglio, M 580

Turley, H 94

Turner, J 60

Turner, T 366

Turpin, FL 820

Tyldesley, S 961

Tzai, T-S 1906

Uchitomi, Y 2089

Udou, T 1098

Ueno, M 1231

Ueno, Y 1690

Ugurel, S 1398

Underwood, MA 625

Uner, A 639

Upadhyay, S 815

Uro-Coste, E 747

Urruticoechea, A 475

Utsunomiya, T 1754

Uzawa, K 1915, 2181

Uziel, O 1881

Vale, C 811

Valentine, HR 449

Valle, JW 628

Valmary, S 743

van Asperen, CJ 1336

Van Belle, S 1055

van Beusechem, VW 882

Van Cutsem, E 1055

Van de Putte, M 1406

van de Velde, CJH 1767

van den Brandt, PA 1310

van den Brule, AJC 1388

van der Meulen, IH 882

van der Meulen, J 797

van der Neut, R 1078

van der Velden, P 2032

van der Vijgh, WJF 1636

van Dulmen, AM 1671

van Groeningen, CJ 1636

van Krieken, JHJM 1767

van Kuppevelt, TH 2249

Van Laethem, J-L 1055

van Muijen, GNP 2249

van Nieuwpoort, F 2032

van Valen, F 705

Vandenheede, JR 1406

Vanichkin, A 294

Vasaturo, T 2225

Vaughan, T 1430

Veal, GJ 696

Vecchia, CL 580

Velten, M 459, 1842

Venet, D 1545

Vennart, W 1599

Vercelli, M 188

Veronese, ML 1846

Verrill, M 475, 1989

Verschraagen, M 1636

Vesovic, Z 1159

Vettorazzi, M 156, 1815

Vicario, G 188

Viglietto, G 1817

Vile, R 1450

Villalona-Calero, MA 1922

Villar, R 690

Villette, J-M 236

Vincenzi, B 2225

Vintermyr, OK 895

Visvikis, D 1046

Vitarelli, S 188

Voest, EE 729

Vogel, T 1126

Vogel, W 1159

Vogten, JM 729

Volkmer, BG 2018

von Delius, S 2129

von Kerczek, A 1430

von Knebel Doeberitz, M 1746

Voorhorst, FJ 1388, 1800

Vormoor, J 705

Vryenhoef, P 1794

Wabinga, H 1808

Wachters, FM 15

Wada, N 847

Wada-Hiraike, O 2286

Wagner, K 1574

Wai, ES 961

Wakiyama, S 1754

Waller, J 265

Waller, M 955

Walsh, G 475

Walters, SJ 55

Walton, M 7

Wander, T 843

Wang, D 1069

Wang, H-C 929

Wang, H-M 30

Wang, S 929

Wang, Y 929, 983, 1414

Wang-Gohrke, S 2039

Ward, C 1976

Ward, TH 532

Wardle, J 265

Wardley, A 1869

Warren, LJ 961

Warrington, A 488

Wartenberg, D 979

Waters, J 1352

Waterton, JC 1599

Wattel, S 1545

Waugh, NR 2070

Webb, DJ 2148

Weber, F 1922

Webster, LR 1366

Wee, J 668, 1382

Weeden, S 1358

Weich, HA 553

Weichert, W 1729

Weiderpass, E 601

Weidle, UH 2249

Weijenberg, MP 1310

Wein, A 2122

Weitzen, R 1611

Welch, CJ 222

Wells, A 366

Wels, WS 1421

Wenger, T 1084

West, CML 449

Westerman, AM 1126

Westerman, M 1636

Westwell, AD 350

Weth, R 1421

Wheelhouse, NM 1268

Whelan, J 1358

White, S 832

Whiteside, TL 209, 913

Widder, J 1209

Wieland, W 843

Wierzchowski, M 1038

Wigmore, SJ 5, 1268

Wikman, FP 2240

Wilander, E 891

Wilkinson, C 60

Williams, D 1892

Williams, ED 2160

Williams, JG 1759

Williams, JR 1531

Williams, L 1759

Williams, MV 2107

Williams, N 1922

Williams, SR 1599

Willis, L 1366

Willits, I 1626

Wilson, BC 298

Wilson, DJ 2148

Wilson, GD 147

Wilson, JHP 1126

Wilson, P 36

Wilton, C 1358

Wipfli, H 1179, 1339

Wiréhn, A-B 1785

Wirkner, U 1934

Wohlberedt, T 1862

Wolfe, MM 1581

Wolff, K 662

Wolk, A 1803

Wöltjen, HH 843

Woods, LM 1279

Woodward, M 2076

Wöppel, A 342

Workman, P 7, 1599

Worley, A 55

Worth, LJ 867

Wotherspoon, A 1352

Wotton, CJ 1298

Wrba, F 1209

Wright, D 147

Wright, JA 1837

Wright, M 2166

Wu, X-C 2084

Wu, Y 729

Wülfing, P 1720

Wyke, SM 711

Xiang, YB 2059

Xie, W-f 607

Xu, CW 1321

Xu, WH 2059

Xu, YC 1321

Yadetie, F 1506

Yagi, H 1737

Yakirevitch, A 1611

Yakobson, E 2278

Yalcin, B 639

Yamada, M 1026

Yamagishi, H 562

Yamaguchi, T 2134

Yamamoto, H 1193

Yamamoto, S 1782

Yamanaka, H 1538

Yamasaki, M 1110

Yamashita, Y 1110

Yanagisawa, A 562

Yang, CH 1013

Yang, RD 1321

Yang, Z 1414

Yano, M 1117

Yano, T 2286

Yao, XT 1430

Yasugi, T 1026, 2286

Yates, C 366

Ychou, M 2114

Yeates, D 1298, 1307

Yee, AS 2153

Yeh, KH 1013

Yerushalmi, R 1881

Yin, Y-H 929

Ylisaukko-oja, SK 1126

Yokoe, H 1915, 2181

Yokomise, H 1231

Yokoyama, M 1240

Yoo, K-Y 1273

Yook, JH 246

Yoon, SY 827

Yoonessi, A 176

Yoshida, A 2216

Yoshida, K 290

Yoshikawa, E 2089

Yoshikawa, H 1026

Yoshimura, A 1711

Younes, MN 1899

Yousaf, U 995

Yu, CS 298

Yu, J 259

Yu, M 334

Yuan, F 1414

Yuklea, M 1517

Zabaglo, L 906

Zahl, P-H 1814

Zalcberg, J 655, 832

Zambon, P 188

Zannoni, GF 271

Zegers, I 1636

Zengin, N 639

Zhan, F 1561

Zhang, C 1084

Zhang, C-Q 929

Zhang, GY 113

Zhang, H 2233

Zhang, L 1430

Zhang, W 983

Zhang, Y 140, 1787, 1942

Zheng, W 2059

Zhong, L 1430

Zhong, W 1493

Zhou, M 2266

Zhu, L 607

Zimmer, Y 41

Zimpfer, A 1398

Zlotnik, Y 294

Zorn, CSM 2018

Zoubek, A 480

Zuidervaart, W 2032

Zummo, G 888

